# Outlines for the Management of the Diet; or, the Regulation of Food to the Requirements of Health and the Treatment of Disease

**Published:** 1887-09

**Authors:** 


Outlines for the Management of the Diet; or, the Regulation of Food to the Requirements of Health and the Treatment of Disease. By Edward Tunis Bruen. Philadelphia: J. B. Lippincott Co. 1887. Pp. 135. Price $1.00.

